# Klotho Regulates 14-3-3ζ Monomerization and Binding to the ASK1 Signaling Complex in Response to Oxidative Stress

**DOI:** 10.1371/journal.pone.0141968

**Published:** 2015-10-30

**Authors:** Reynolds K. Brobey, Mehdi Dheghani, Philip P. Foster, Makoto Kuro-o, Kevin P Rosenblatt

**Affiliations:** 1 Centers for Proteomics and Systems Biology, Brown Foundation Institute of Molecular Medicine, The University of Texas Health Science Center at Houston (UTHealth) Medical School, 1825 Pressler Street, Houston, Texas, 77030, United States of America; 2 Division of Oncology, Department of Internal Medicine, The University of Texas Health Science Center at Houston (UTHealth) Medical School, 6410 Fannin, UTPB Suite 722, Houston, Texas 77030 United States of America; 3 Companion Dx Reference Laboratory, LLC, 10301 Stella Link Rd., Suite C, Houston, Texas 77025, United States of America; 4 Department of NanoMedicine and Biomedical Engineering, The University of Texas Health Science Center at Houston (UTHealth), MD Anderson Cancer Center Bldg-3SCRB, 1881 East Road, Houston, Texas 77030, United States of America; 5 Division of Pulmonary Medicine, Department of Internal Medicine, The University of Texas Health Science Center at Houston (UTHealth) Medical School, 6431 Fannin, MSB 1.274, Houston, Texas 77030, United States of America; 6 Center for Molecular Medicine, Jichi Medical University, 3311–1 Yakushiji, Shimotsuke, Tochigi 329–0498, JAPAN; 7 Department of Pathology, Center for Mineral Metabolism, University of Texas Southwestern Medical Center, 5323 Harry Hines Blvd., Dallas, Texas 75390–9072, United States of America; McGill University Department of Neurology and Neurosurgery, CANADA

## Abstract

The reactive oxygen species (ROS)-sensitive apoptosis signal-regulating kinase 1 (ASK1) signaling complex is a key regulator of p38 MAPK activity, a major modulator of stress-associated with aging disorders. We recently reported that the ratio of free ASK1 to the complex-bound ASK1 is significantly decreased in Klotho-responsive manner and that Klotho-deficient tissues have elevated levels of free ASK1 which coincides with increased oxidative stress. Here, we tested the hypothesis that: 1) covalent interactions exist among three identified proteins constituting the ASK1 signaling complex; 2) in normal unstressed cells the ASK1, 14-3-3ζ and thioredoxin (Trx) proteins simultaneously engage in a tripartite complex formation; 3) Klotho’s stabilizing effect on the complex relied solely on 14-3-3ζ expression and its apparent phosphorylation and dimerization changes. To verify the hypothesis, we performed 14-3-3ζ siRNA knock-down experiments in conjunction with cell-based assays to measure ASK1-client protein interactions in the presence and absence of Klotho, and with or without an oxidant such as rotenone. Our results show that Klotho activity induces posttranslational modifications in the complex targeting 14-3-3ζ monomer/dimer changes to effectively protect against ASK1 oxidation and dissociation. This is the first observation implicating all three proteins constituting the ASK1 signaling complex in close proximity.

## Introduction

Human aging is a multi-faceted process influenced by both genetic and environmental factors. Although studies alluding to the genetic basis of aging have been reported extensively [1,2], the discovery of Klotho an anti-aging protein hormone [3] over a decade ago has further refurbished our understanding that aging can also be controlled by humoral factors. Since then, Klotho has been linked to multiple functions including inhibition of insulin/insulin growth factor1 (IGF1) signaling, regulation of calcium/phosphate metabolism, as an obligate co-receptor for fibroblast growth factor 23 (FGF23), and a pathological role as tumor suppressor in cancer [4–6]. Furthermore, lower expression levels of Klotho in the brain white matter of non-human primates have been linked to neurological disorders [7]. And more recently, publications detailing Klotho’s protective role in the brain have emerged [8–11]. Yet, the molecular basis underlying Klotho functions remains largely unknown. One remarkable feature pertaining to Klotho overexpressing cells and tissues is their relatively lower oxidative status, while the reverse is true for Klotho deficient systems where oxidative stress levels are much higher [3,12]. These data suggest that Klotho activity exhibits cross-talk with pathways that control oxidative stress levels. It has been established that endogenous reactive oxygen species (ROS) produced by mitochondrial electron transport chain (ETC) dysfunction activate p38 MAPK, which is a major contributor to stress-associated aging disorders in different aging models [13–15]. This pathway is activated through the apoptosis signal-regulating kinase 1 (ASK1) signaling complex. We previously reported that the p38 MAPK activity in the livers of Klotho overexpressing and Klotho deficient mice is regulated by ROS-sensitive ASK1 signaling complex [16].

Existing theories that describe ASK1 dissociation and activation all rely exclusively on redox interactions of Trx with the signaling complex [17,18]. Whereas Trx is a key signaling molecule among proteins in the ASK1 activation pathway identified to date, the discovery of Klotho’s involvement in this pathway has necessitated the search for the role played by other proteins in the complex. In this study, we tested our hypothesis for Klotho-ASK1 regulation that: 1) covalent interactions exist among three identified proteins constituting the ASK1 signaling complex; 2) in normal unstressed cells the trio ASK1, 14-3-3ζ and thioredoxin (Trx) simultaneously engage in a tripartite complex formation; 3) Klotho’s stabilizing effect on the complex relied solely on 14-3-3ζ expression and its apparent dimerization changes. In addition, we provide an alternative model describing ASK1 complex formation and dissociation and propose specific role for Klotho signaling in resistance to oxidative stress.

## Materials and Methods

### Cell culture

The Klotho responsive HEK 293 cells used in this study were routinely maintained in Gibco’s Dulbecco’s Modified Eagle Medium (DMEM) with 4.5 g/L glucose, 2 mM glutamine and 1 mM sodium pyruvate (Life Technologies, Carlsbad, CA), supplemented with 10% fetal bovine serum and 100 μg/ml penicillin/streptomycin. Cells were pretreated with either 200 pM recombinant secreted Klotho obtained as described [12] or 20 mM *N*-Acetyl cysteine (NAC) for 30–40 min before challenge with 5 μM rotenone (ROT) (Sigma, St Louis, Mo) for an additional 40 min. Harvested cells were washed three times with cold phosphate buffered saline (PBS) containing 50 mM sodium fluoride (NaF) and 2 mM sodium orthovanadate (Na_3_VO_4_) and kept at -80°C until use.

### Protein preparation

Clear lysates were prepared using the ProteoExtract Cytosol/Mitochondria Fractionation Kit (EMD Biosciences, San Diego, CA). All centrifugations were done at 4°C. The lysis buffer was supplemented with phosphatase inhibitors where needed. The protein concentration of the samples was determined using the Bradford Assay Kit (Bio-Rad, Hercules, CA).

### siRNA

14-3-3ζ Stealth siRNA duplex oligoribonucleotides (three 14-3-3ζ-targeted sequences) and control siRNAs were purchased from Life Technologies (Carlsbad, CA). Cells were seeded in 6-well culture dishes and transfected with 100 nM of the pooled siRNAs using Lipofectamine 2000 as carrier molecule.

### Immunoprecipitation

Immunoprecipitation (IP) assays were performed as follows: approximately 200–400 μg proteins from clear lysates were incubated with 1–2 μg of the antibody for 6–7 h in IP buffer (20 mM Tris, pH 7.5, 150 mM NaCl, 2 mM EDTA, and 1 mM EGTA) supplemented with protease and phosphatase inhibitor cocktails at 4°C with gentle agitation. Approximately 30 μl (bed volume) of protein A-conjugated agarose beads were added and incubation resumed for additional 4 h.

### SDS-PAGE and Western blot

IP samples were processed for SDS-PAGE and Western blot as follows: beads were washed five times with the IP buffer described above, resuspended in 50 μl of sample loading buffer and separated on 4–12% NuPAGE gels (Life Technologies). Where no heating was necessary, samples were incubated with loading buffer for 30 min at room temperature under reducing and/or non-reducing conditions. The resolved proteins were electroblotted onto PVDF membranes (GE Healthcare) using the XCell II Blot Module (Life Technologies). Transfer was conducted constantly at 30 V for 1 h. Blots were incubated with appropriate primary and secondary antibodies and protein signals were detected with the Novex^R^ ECL Western blotting detection reagents (Life Technologies). Where protein phosphorylation levels were measured, specific anti-phosphorylation primary antibodies were used. Blots were then stripped using Restore Western Blot Stripping Buffer (Thermo Scientific/Pierce, Rockford, IL) and re-probed with the desired antibodies for total protein detection. The signal intensity of phosphorylated bands was normalized with that of the total protein and the average ratios were plotted with standard deviations.

### DNA transfection and GST pull-down assay

Mammalian plasmids expressing the GST-tagged 14-3-3ζ wild type (Wt) and the dimer deficient mutant (Dm) were originally constructed by the Avruch laboratory group [19] or were purchased from Addgene (Cambridge, MA). Approximately 4 μg DNA was transfected into 293 cells (2.5x10^5^ cells per well) using an established protocol with Lipofectamine 2000 as a carrier. Cells were harvested 48 h post-transfection and stored at -80°C until needed. Total proteins were prepared from cells as described above. Equal amounts of protein from Wt and Dm lysates were adsorbed onto glutathione sepharose beads (GE Healthcare, Piscataway, NJ), washed five times with binding buffer (20 mM Tris-Cl pH 7.5, 2 mM EDTA, 1 mM EGTA and 80 mM NaCl, supplemented with protease inhibitor cocktail) and eluted into SDS sample buffer. SDS-PAGE and Western blot were all performed as described above in the Materials and Methods.

### Immuno-capillary electrophoresis

The iCE utilizing NanoPro CB1000 platform (ProteinSimple, Santa Clara, CA) was performed on co-IP complexes with ASK1 antibody from Klotho-treated cells and untreated controls to support studies on the complex interactions in the lysate while still in the native state. The NanoPro platform is a fully automated liquid phase capillary fractionation technology that separates complex mixtures of proteins in the nanogram range utilizing the proteins’ native isoelectric point (*p*I) chemistries. The following parameters were optimized for the assay as follows: protein mixture working concentration was 0.025 mg/ml; primary antibody dilutions were 1:33 for anti-Trx, 1:50 for anti-ASK1 and 1:250 for anti-14-3-3ζ; and, the secondary antibody (rabbit IgG-HRP) was diluted at 1:100. All reagents used were strictly obtained from the manufacturer and data analyses were carried out using the integrated software (Compass^TM^) also provided by the manufacturer. Samples were run in replicates and the experiments were repeated at least twice. The patterns were reproducible, but the absolute values between replicated varied widely; thus, only representative graphs are displayed in the figures.

### Non-reducing 2D-GE

Non-reducing, two-dimensional gel electrophoresis (2D-GE) Western blots were performed on Klotho-treated cells and untreated controls to investigate whether intrinsic disulfide-bridges are relevant in the complexes formed with Trx, ASK1 and/or 14-3-3ζ and to further determine whether Klotho promotes this interaction. Cells grown to appropriate confluence were freeze-precipitated with 10% trichloroacetic acid (TCA), the so-called acid trap, to discourage further thiol-disulfide exchange reactions. The precipitate was washed five times with cold acetone to adjust for low pH. Precipitated cells were then further denatured in 6M GuHCl buffer containing 50 mM iodoacetamide for 2 h to alkylate available free thiol groups. Following cleansing by precipitation, protein pellets were re-constituted in conventional urea buffer for running 2D-GE analyses, but strictly under non-reducing conditions. Resolved proteins were Western transferred onto PVDF membranes and probed with Trx, ASK1, or 14-3-3ζ antibodies as described above.

### Statistical analysis

Statistical analyses were performed using *SYSTAT*, version 8. Data were analyzed using repeated-measures ANOVA, and paired t tests. For all pairwise comparisons, the *P*-values were adjusted by using post hoc Bonferroni and Dunn-Sidak corrections to *P*-values to adjust for multiple testing. After correction, if the new adjusted threshold of *P*-value significance is less than 0.05, the observed difference is considered statistically significant and hence evidence against the null hypothesis of zero mean difference.

## Results

### Secreted Klotho mitigates oxidant-induced Trx/ASK1 complex dissociation and suppresses downstream activation of p38 MAPK in cultured cells

**Fig 1:**
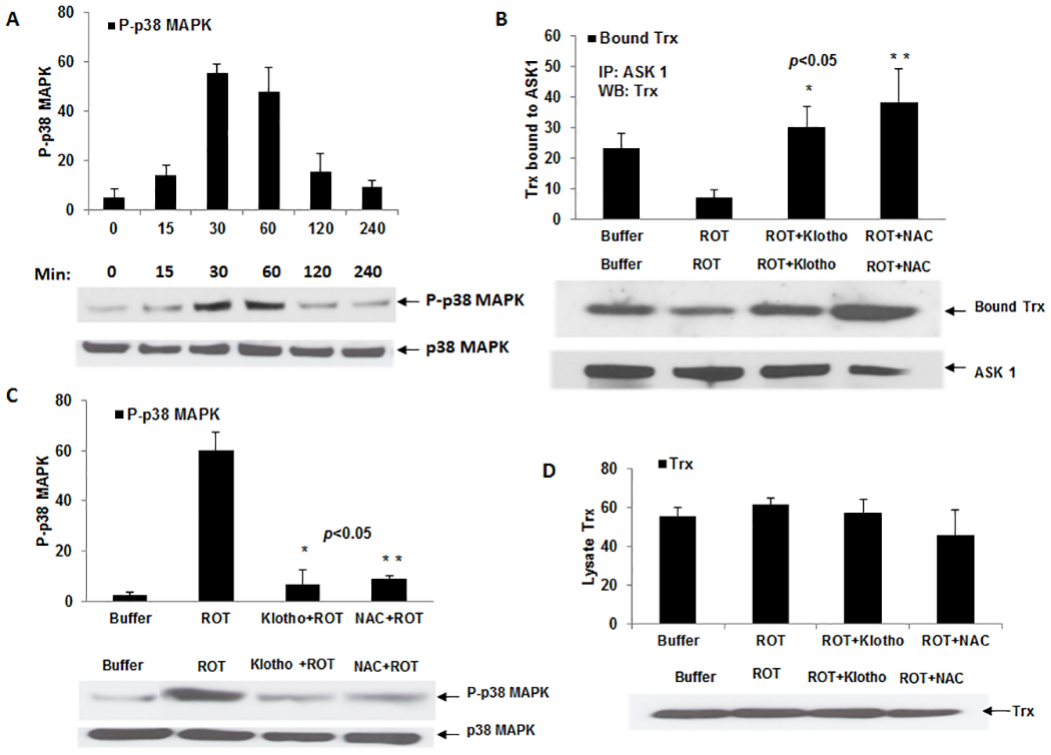
The effects of secreted Klotho on rotenone (ROT)-induced activation of p38 MAPK and Trx/ASK1 complex levels in HEK 293 cells. (A) Plots of time course of ROT-mediated activation of p38-MAPK. Experiments were done at least twice and deviations are shown ± SEM. A representative Western blot is shown beneath plot. (B) Depiction of Trx/ASK1 complex levels in HEK 293 cells after pre-incubation with either control buffer alone or 200 pM Klotho/20 mM NAC for 40 min before adding the 5 μM ROT to induce oxidative stress. ASK1 was immunoprecipitated (IP) with rabbit polyclonal ASK1 antibody and the co-precipitated Trx was revealed by Western blot. A representative Western blot of the samples is shown beneath. *p< 0.05 between ROT and Klotho + ROT. (C) Plots of p38 MAPK activation levels in HEK 293 cells treated with either Klotho or NAC as described in section B, with a representative Western blot. *p< 0.05 between ROT and Klotho + ROT. (D) Total lysate levels of Trx prior to IP. Where phosphorylation was studied, the same blot was stripped with Restore^TM^ stripping buffer (Thermo Scientific) and re-probed with total p38 MAPK antibody for protein normalization. Digitized values of the WB of are shown in S1 and S2 Tables.

Previous study has unambiguously described regulatory role for Klotho protein via the ASK1/p38 MAPK signaling pathway [16]. To provide further mechanistic insight into Klotho’s activity on signaling proteins in the ASK1/p38 MAPK pathway, we first developed a cell-based stress response assay by stimulating HEK 293 cells with 5 μM rotenone (ROT), which generates ROS via specific mitochondrial electron transport chain dysfunction (ETC) as previously reported [16]. Levels of p38 MAPK activation were measured using Western blot analysis. The result shown in Fig 1A indicates p38 MAPK activation in HEK 293 cells is dynamically regulated between 0–2 h, peaking between 30–60 min, and decreasing in intensity thereafter.

Next, using 40 min as the target for measuring activation of p38 MAPK in HEK 293, we determined endogenous Trx/ASK1 complex levels in ROT-treated cells verses buffer-treated controls by co-immunoprecipitating (co-IP) ASK1 with anti-ASK1 antibody and probing for co-precipitated Trx by Western blot analysis. As shown in Fig 1B, the levels of immunoprecipitated ASK1 revealed using an ASK1 antibody remained relatively unchanged for both ROT-treated cells and the untreated control [95% CI: 7.828–22.239, Bonferroni-adjusted *P*-value = 0.073, Dunn-Sidak-adjusted *P*-value = 0.071]. By contrast, the level of bound Trx detected from the Trx/ASK1 complex in ROT-treated cells was significantly lower (p<0.05) compared to the buffer-treated controls.

With our stress response assay in place, we then tested whether purified Klotho added exogenously to cultured cells protects the Trx/ASK1 complex from ROT-induced dissociation. We pre-incubated HEK 293 cells with 200 pM Klotho for at least 40 min before re-treating them for another 40 min with 5 μM ROT. The concentration of Klotho and pre-incubation time used here were predetermined based on our previous report [12]. The relative Trx/ASK1 complex levels in ROT-treated verses buffer-treated cells correlated with the downstream activation status of p38 MAPK (Fig 1C) [95% CI: (- 72.099)–(-35.434), Bonferroni-adjusted *P*-value = 0.037, Dunn-Sidak-adjusted *P*-value = 0.037]. Thus, the Klotho [95% CI: 40.669–60.797, Bonferroni-adjusted *P*-value = 0.013, Dunn-Sidak-adjusted *P*-value = 0.013] or NAC pre-treated cells [95% CI: 30.970–64.697, Bonferroni-adjusted *P*-value = 0.040, Dunn-Sidak-adjusted *P*-value = 0.039] exhibited reduced p38 MAPK activation, in contrast to the increased activation observed in ROT only-treated cells (Fig 1C). We observed no significant difference between Klotho and NAC pre-treated cells [95% CI: (-11.298) to—5.498, Bonferroni-adjusted *P*-value = 1.000, Dunn-Sidak-adjusted *P*-value = 0.856]. Total Trx levels in the starting lysates remained unchanged between ROT-treated and buffer-treated cells (Fig 1D). Moreover, Fig 1B indicates, pre-incubating cells with secreted Klotho significantly protected the endogenous Trx/ASK1 complex from ROT-induced dissociation [95% CI: (-32.637)—(-10.096), *P*-value = 0.015], comparable to the protection exhibited by the well-known ROS scavenger *N*-acetyl cysteine (NAC) (similar, *P*-value > 0.05) (Fig 1B).

### Secreted Klotho increases the 14-3-3ζ phosphorylation, monomer levels and interaction with Trx in cultured cells

**Fig 2:**
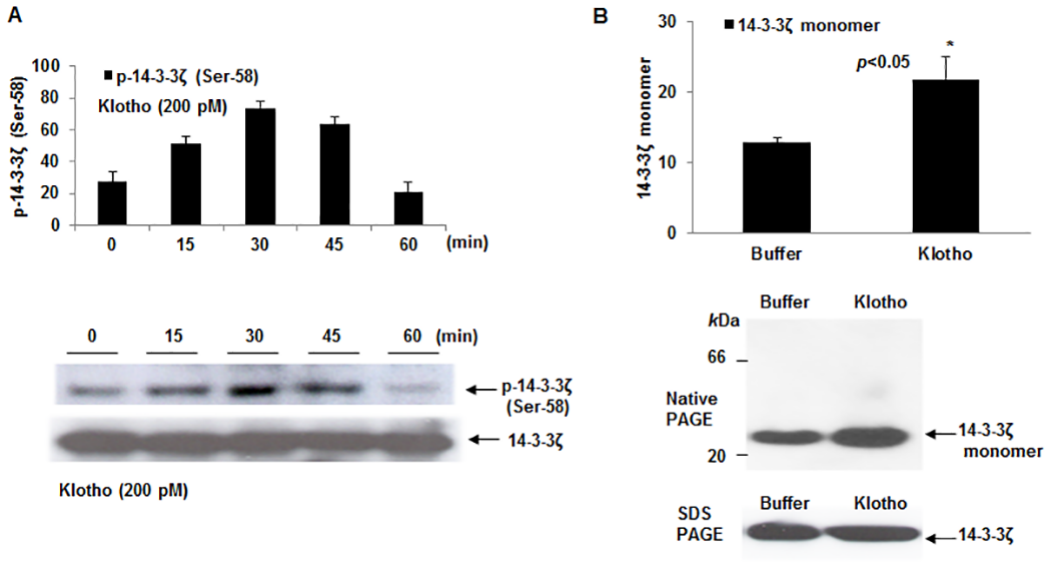
Time course of Klotho-mediated phosphorylation of endogenous 14-3-3ζ (Ser-58) effect on 14-3-3ζ monomer levels in HEK 293 cells. (A) Plot describing time-dependent 14-3-3ζ phosphorylation mediated by Klotho was demonstrated by adding 200 pM of the recombinant protein to the cultured cells at the indicated times. Peak phosphorylation times (i.e. 30–45 min) are within the range where the Trx/ASK1 complex protection against oxidative stress occurred (Fig 1). Data are reported as ± SEM. (B) Plot of 14-3-3ζ monomer level in HEK 293 cells treated with either secreted Klotho or buffer control for 40 min. Shown beside plot is a native Western blot of the samples as described in Materials and Methods. The 14-3-3ζ antibody recognizes a predominant ~30 *k*Da protein band representing the expected size of the monomer. Replicate samples were separated under SDS-PAGE, electroblotted onto PVDF membrane and probed with same antibody to account for lysate levels of total 14-3-3ζ. Digitized values of the WB of monomer levels are shown in S3 Table.

The 14-3-3 proteins are components of the ASK1 signaling complex. To investigate whether Klotho activity impacts on 14-3-3ζ structure and interaction with client proteins in the complex, we cultivated HEK 293 cells with or without 200 pM Klotho at various time points and analyzed the level of 14-3-3ζ phosphorylation on Western blots. The results obtained (Fig 2A) clearly indicate that Klotho triggers phosphorylation of 14-3-3ζ as a time-dependent function, reaching a maximum between 30–45 min, and diminishing in intensity thereafter, suggesting an active regulation by specific kinase(s) and phosphatase(s).

Since phosphorylation of 14-3-3ζ at Ser-58 is thought to regulate 14-3-3ζ monomer/dimer changes [20,21] due to its location in the dimer interface [22,23], we attempted to establish whether the Klotho signaling effect on phosphorylation of the endogenous 14-3-3ζ may also regulate its monomer/dimer levels. In addition, we wanted to confirm whether the relatively higher 14-3-3ζ monomer levels observed in Klotho overexpressing mice (see companion paper) is actively duplicated in the cell. To do this, we performed native PAGE on lysates of Klotho-treated and buffer only-treated HEK 293 cells. Separated proteins were then transferred to membranes and probed with an anti-14-3-3ζ antibody for detecting the presence of native 14-3-3ζ molecular forms. As shown in Fig 2B, the data indicate an increased accumulation of a ~ 30 *k*Da protein on Western blot, which represents an increase in the monomeric 14-3-3ζ form. For an unbiased estimation of total 14-3-3ζ in the cell lysates, we performed a SDS-PAGE Western analysis on replicate samples from treated cells and observed no significant changes in total 14-3-3ζ protein (shown beneath Fig 2B native blot).

**Fig 3:**
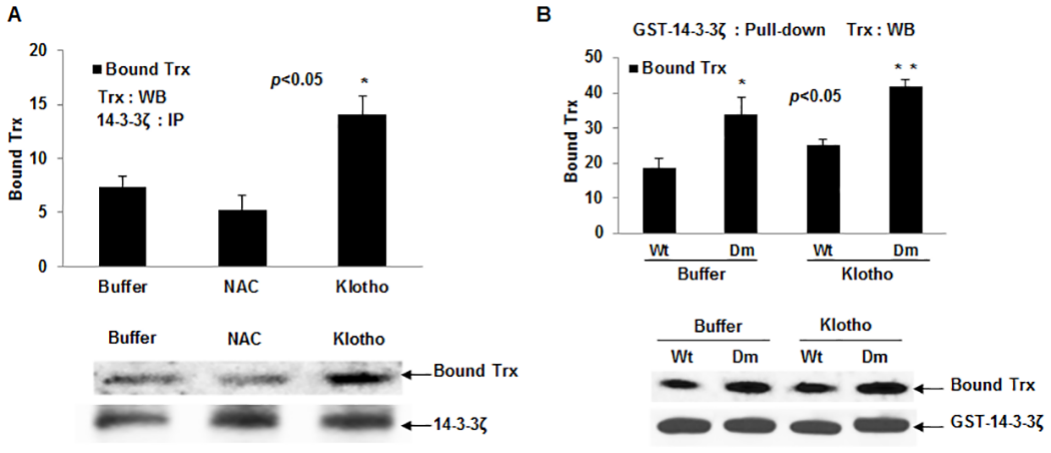
Mitigating effect of soluble Klotho on endogenous 14-3-3ζ and Trx complex formation. (A) Levels of Trx bound to endogenous 14-3-3ζ in HEK 293 cells treated with either secreted Klotho, or NAC, or buffer control for 40 min as described in text. *p< 0.05 between control buffer and Klotho treated samples. The Western blot profile is shown beneath. (B) Levels of Trx bound to GST-14-3-3ζ wild type (Wt) and dimerization deficient mutant (Dm). Samples were treated with either buffer only or secreted Klotho for 40 min. *p< 0.05 between Wt and Dm. Shown beneath is a corresponding representative Western blot. Digitized values of the WB are shown in S4 Table.

Since 14-3-3 monomer/dimer changes have been linked to the specification of 14-3-3-client protein interactions, we hypothesized that Klotho-mediated Ser-58 phosphorylation will influence the Trx/14-3-3ζ complex interaction. To test this hypothesis, we performed IP with a 14-3-3ζ antibody using cells treated with Klotho or NAC, or with control buffer for 40 min, and ran Western blot to estimate the levels of complex-bound Trx (Fig 3A). An approximately 2-fold increase in levels of bound Trx was observed for Klotho-treated cells verses buffer only-treated control cells (p<0.05). By contrast, no apparent increase in bound Trx was found for NAC-treated cells (Fig 2C) implying that the Klotho effect depended on a different specific signaling event.

For further demonstration that Trx may preferentially bind to the monomeric 14-3-3ζ, we artificially expressed a glutathione-*s*-transferase (GST)-tagged 14-3-3ζ wild type (14-3-3ζwt) protein and its dimer deficient mutant (14-3-3ζdm) in HEK 293 cells and analyzed the cell lysates for co-precipitated Trx as before. As indicated in Fig 3B, an approximate 2-fold increase in bound Trx was observed in 14-3-3ζdm expressing relative to 14-3-3ζwt expressing cells; this effect was further increased when transfected cells were pretreated with secreted Klotho for 40 min.

### 14-3-3ζ depletion increases ROS-mediated p38 MAPK activation irrespective of Klotho action

We determined that incubation of recombinant secreted Klotho with HEK 293 cells induced 14-3-3ζ phosphorylation, which was revealed with a Ser-58-specific antibody, and inferred that such phosphorylation could be a key posttranslational modification producing 14-3-3ζ interactions with the ASK1 signaling complex. At the same time, our cell-based assays demonstrated that pre-treatment of HEK 293 cells with Klotho prior to inducing oxidative stress with ROT significantly protected against Trx/ASK1 complex dissociation.

**Fig 4:**
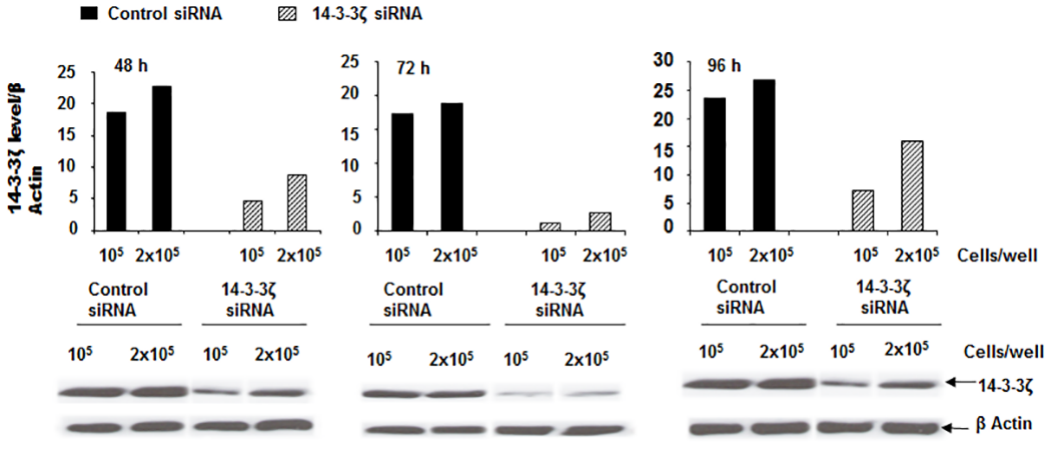
siRNA knockdown of 14-3-3ζ expression in HEK 293 cells. A time dependent plot of levels of 14-3-3ζ expression following siRNA knockdown of the 14-3-3ζ in HEK 293 cells. Cells were seeded at either 1.0 or 2.0 x10^6^cells/well in 6-well culture dishes. For 14-3-3ζ targeting, three independent Stealth RNA duplex primers (Life Technologies) were pooled and used at a final concentration of 100 nM. Residual 14-3-3ζ expression levels were determined at 48, 72 and 96 h post transfection. Beneath the plots are Western blots showing the extent of 14-3-3ζ depletion (optimal at 72 h) at 1.0 x 10^6^ cells/well.

**Fig 5:**
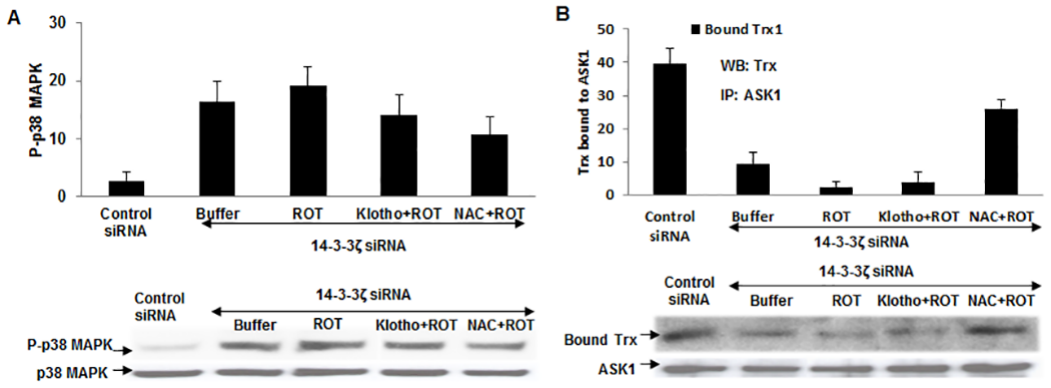
Effect of 14-3-3ζ knockdown on Klotho signaling via ASK1 and p38 MAPK pathway. (A) Plots of phosphorylation levels of p38 MAPK in 14-3-3ζ-depleted HEK 293 cells. Cells were pre-treated with either 200 pM secreted Klotho or 20 mM NAC for 40 min before the addition of 5 μM ROT. Level of the phosphorylated protein was measured by Western blot using a phospho-14-3-3ζ (Ser-58) antibody. The same membrane was stripped and re-probed with the total 14-3-3ζ antibody to normalize for protein load. (B) Levels of Trx bound to ASK1 in 14-3-3ζ-depleted HEK 293 cells treated with secreted Klotho, or NAC, or buffer control for 40 min as described in text. The Western blot profile is shown beneath.

To find out the extent of 14-3-3ζ contribution to Klotho mediation, we performed knockdown 14-3-3ζ expression assays in HEK 293 cells by siRNA using appropriate controls (Fig 4). Cells were then cultivated with or without 5 μM ROT and/or 200 pM Klotho and assayed for both p38 MAPK phosphorylation and Trx/ASK1 complex levels (Fig 5A and 5B). As shown in Fig 4, approximately 80–90% of 14-3-3ζ protein expression was depleted when cells were seeded at 1 x 10^5^ cells/9.6cm^2^ dishes and re-cultured for 48 h post-transfection.

Using the 14-3-3ζ-knockdown cells in subsequent experiments, Klotho activity in the presence of reduced expression of 14-3-3ζ did not effectively prevent the Trx/ASK1 complex dissociation induced by 5 μM ROT; however, the ROS scavenging activity of NAC was only moderately affected (Fig 5B). This result suggests Klotho mediation via a specific pathway rather than a non-specific anti-oxidant effect. Moreover, even without the ROS induction, 14-3-3ζ-knockdown cells showed increased basal p38 MAPK phosphorylation (Fig 5A), which is consistent with a previous report [24]. Pre-treating 14-3-3ζ-knockdown cells with Klotho did not significantly reduce the p38 MAPK phosphorylation levels while NAC was moderately effective (Fig 5A). These observations strongly suggest that 14-3-3ζ plays a key role in the ASK1/p38 MAPK, stress-response pathway affected by Klotho.

### Non-reducing 2D-GE Western blot reveals the necessity for disulfide-bridging

**Fig 6:**
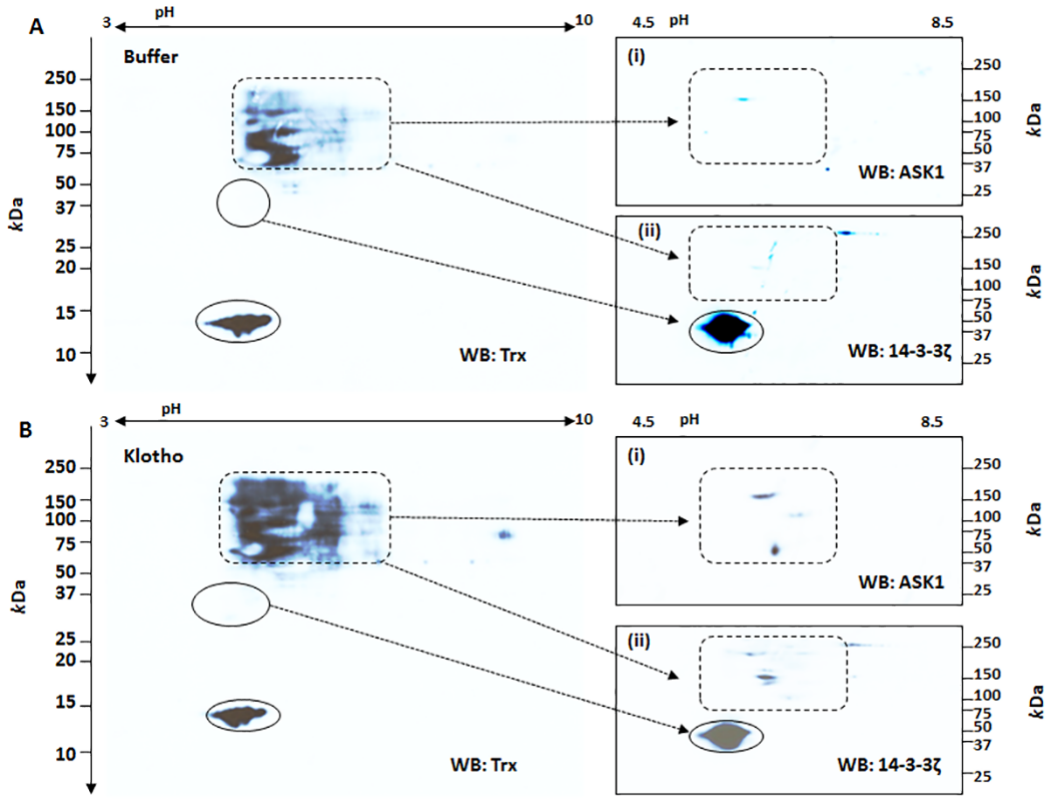
Non-reducing 2D-GE Western blot analyses of ASK1 signaling complex in HEK 293 cells. A non-reducing 2D-GE that preserves endogenous disulfide linkage was performed on Klotho-treated and buffer-control cells as described in the text. Images in A (buffer treated) or B (Klotho treated) are representative Western blots probed with either Trx antibody or stripped and re-probed with (i) ASK1 and/or (ii) 14-3-3ζ. Closed circled spots indicate free Trx and/or 14-3-3ζ proteins based on *p*I and/or molecular size. The dotted rectangular spots represent ASK1-bound complexes with Trx and/or 14-3-3ζ and other proteins cross-reactive with the Trx antibody that were more abundant in Klotho treated cells than in the buffer control.

Although Trx/ASK1 complex formation [17,18] and, more recently, Trx/14-3-3 interaction [25] are described as redox-sensitive, studies thus far have not been clear on the nature of forces that govern these interactions [14,17,26,27]. To clarify the importance of covalent interactions against the requirement of a non-covalent one, we performed non-reducing 2D-GE Western blot analyses to test the importance of intrinsic disulfide bridging; we further intended to resolve whether Klotho activity promotes these interactions. Cell lysates were prepared in a way that preserves endogenously formed mixed disulfide bridges [28]. Blots were initially probed with the Trx antibody to reveal size- and *p*I-matched free Trx (full rectangle) and disulfide-linked, and complex-bound, Trx (dotted rectangle) (Fig 6A and 6B). The same membranes were then stripped and re-probed with (i) ASK1 and (ii) 14-3-3ζ antibodies. The ASK1 antibody revealed a single, predominant signal between 150–250 *k*Da with a *p*I of ~5.8–6.1 in the buffer-only, control blot, while the Klotho-stimulated blot uncovered multiple signals, one of which has a similar size and *p*I as the control, but with higher intensity (Fig 6B i). The 14-3-3ζ antibody on the other hand, gave several distinct signals on the blots (Fig 6A and 6B ii), yet more intense in the Klotho-stimulated cells than the -unstimulated control. These signals represent both free 14-3-3ζ (25–37 *k*Da; *p*I ~4.9) and complexes formed with disulfide-dependent client proteins (100–250 *k*Da; *p*I 5.2–6.6) (Fig 6A and 6B ii). The complex-bound 14-3-3ζ is mostly within the size and *p*I ranges of ASK1 described above, implying possible intermolecular associations and that the Klotho effect increases the interactions. Indeed, the above data were further supported in subsequent analyses of the complex interactions described below.

### Disulfide reducing agents are required to effectively dissociate Trx from complexes formed with ASK1 and/or 14-3-3ζ

**Fig 7:**
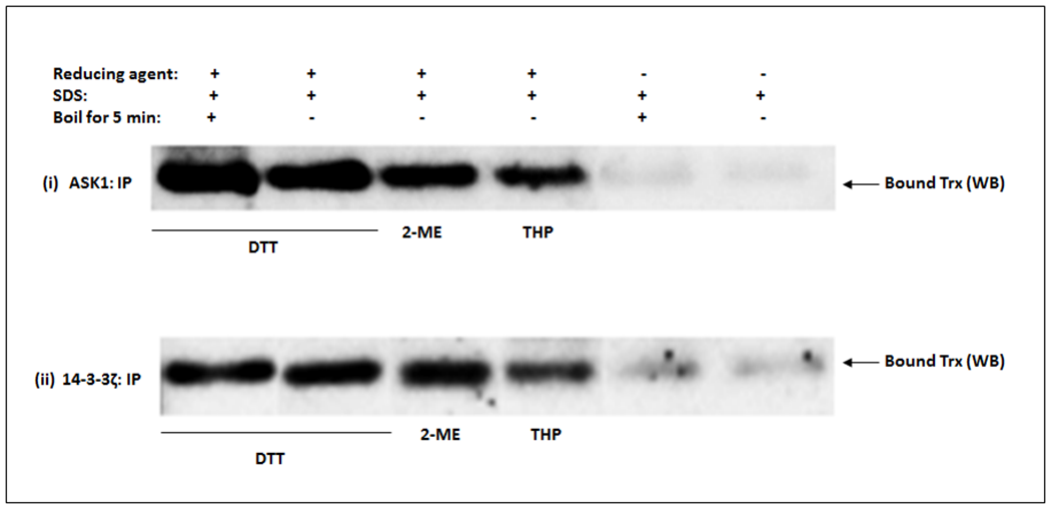
Effect of disulfide reducing agents on the dissociation of Trx from Trx bound ASK1 and/or 14-3-3ζ complex. The ASK1 signaling complex was co-IP using ASK1 or 14-3-3ζ antibody. Chemical dissociation of co-precipitated Trx from the complex was monitored in the presence or absence of various disulfide reducing agents in SDS- conditioned buffer as described in the text. DTT, dithiothreitol; 2-ME, 2-mercaptoethanol; THP, tris(hydroxypropyl)phosphine. The experiment was repeated at least twice and a representative Western blot is shown. A representative full blot image showing higher molecular weight protein complexes is shown in S1 Fig

To strengthen our observation regarding the covalent forces that hold the complex together as described earlier on, co-IP Trx/ASK1 and/or Trx/14-3-3 complexes were immobilized onto an agarose support and dissociation of Trx from the complex was monitored in the presence or absence of various disulfide reducing agents. Our results (Fig 7) show that where a disulfide reducing agent was omitted, there was essentially no dissociation of Trx from the complex; even when the complexes were boiled for 5 min in the presence of SDS. By contrast, inclusion of 50 mM of any one of the tested thiol reagents, namely DTT, 2-ME or THP, resulted in appreciable dissociation of Trx from the complexes, irrespective of whether samples were incubated at room temperature for 20 min or boiled for 5 min (Fig 7). The fact that thiol-based reducing agents are required to effectively dissociate Trx from the complex suggests that the predominant chemical force binding endogenous Trx to ASK1 and/or 14-3-3ζ may involve the formation of mixed disulfide bridges and reinforces the previous observation that double mutations of the two active site cysteine residues of Trx abolished its interaction with ASK1 completely but a single mutation does not [29].

### Immuno-capillary electrophoresis assay (iCE) reveals that Trx/14-3-3ζ/ASK1 interactions can occur in a single complex

**Fig 8:**
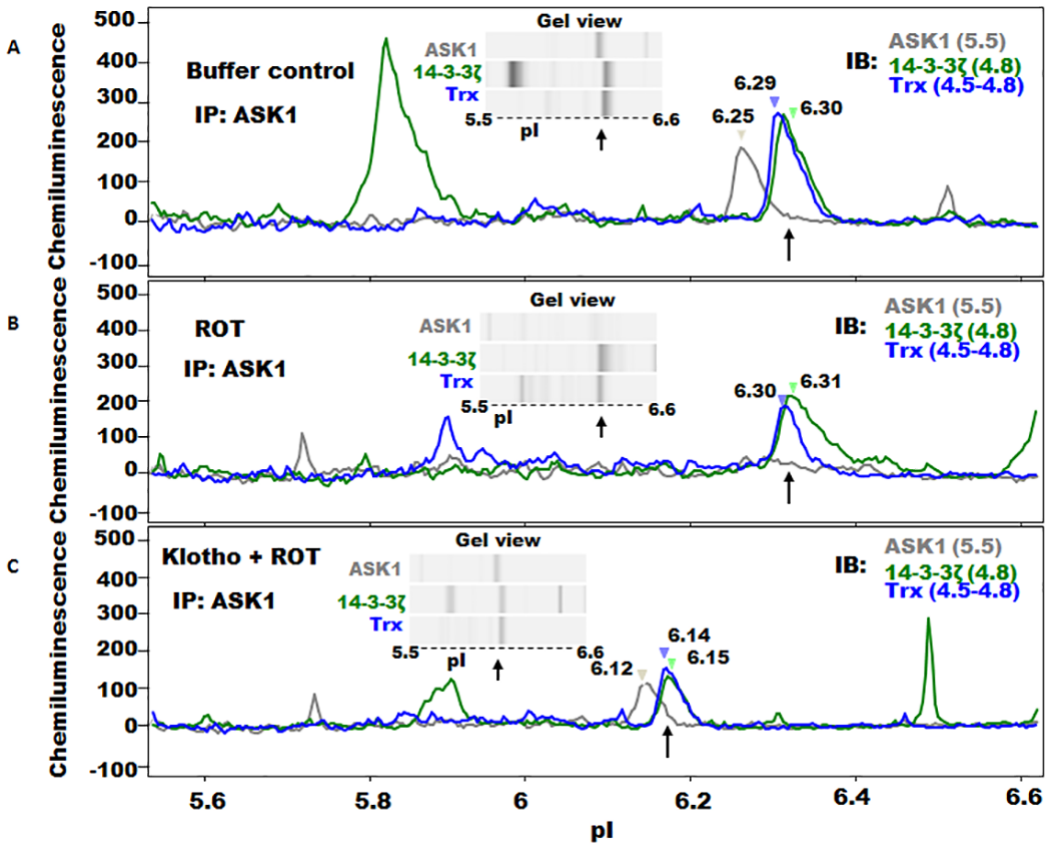
iCE analyses of ASK1 signaling complex interactions. Cells were either pretreated with buffer only or secreted Klotho for 40 min before adding 5 μM ROT to induce oxidative stress. Clear lysates were co-IP with antibody against ASK1 and the eluted protein complex was subjected to immuno-CE fractionation as described in the text. (A) Immuno-CE profile of eluted complex of buffer-only-treated control cells originally separated within pH 4–8 (*p*I range 5.5–6.6 is shown here for clarity) showing overlapping protein signals (arrow) within the *p*I range 6.25–6.30 comprising the ASK1/14-3-3ζ/Trx complex assembly. Signals belonging to individual proteins dissociated from the triad were also found within the pH 4–8. Embedded is a software generated gel view of the complex within the specified *p*I range. Individual *p*Is of the proteins are indicated in parenthesis. (B) Profile of eluted complex from cells treated with ROT. Overlapping signals within *p*Is 6.29–6.30 (arrowed) are those of the 14-3-3ζ/Trx complex. ASK1 signal was undetectable in the presence of ROT. Also shown is the gel view within the *p*I 5.5–6.6 range. (C) Profile of eluted complex from cells pretreated with secreted Klotho prior to ROT treatment. Overlapping signals within the *p*I 6.12–6.15 range (arrow) are the protected triad ASK1/14-3-3ζ/Trx complex. Shifts in *p*I of the complex to acidic end are noticeable.

The ability to analyze interactions of the ASK1 signaling complex on a single platform while still in their native state will provide some clue to their spatial arrangement. To do this, we used iCE (ProteinSimple), which is a capillary-based immuno-separation technology that resolves complex mixtures of proteins, in nanogram quantities, based on their isoelectric point (*p*I) while still in their native state. For the purpose of this study, we used antibodies to all three proteins in a common platform and looked for common peaks in each separate immunoassay to delineate how the complex orients in response to oxidative stress, and whether secreted Klotho has any effect on this arrangement. To facilitate enrichment of the complex for reliable immuno-detection, we first co-precipitated the complex via the ASK1 antibody as described earlier, and then eluted the proteins before running the assays. Complexes from the various cell treatments described below were saved as protein fractions. The results of the analyses are shown in Fig 8A–8C. In the buffer control fraction shown in panel A, an endogenous ASK1 forms complexes with Trx and 14-3-3ζ and is seen as three overlapping signals within a resolution of 0.05 *p*I units (Fig 8, arrows at *p*I 6.25–6.30). Although protein signals were detected within the entire pH 4–7 range, only those within the *p*I 5.5–6.6 surrounding the ASK1/14-3-3ζ/Trx complex signals are shown for clarity. Embedded in the graph is a software-generated gel view of the complexes displaying positioning of the various signals. By contrast, complexes formed in the presence of ROT (panel B) revealed reduced signal levels of Trx and 14-3-3ζ with complete dissociation of ASK1 from the complex (Fig 8B, arrow); under this condition, however, 14-3-3ζ still remained bound to Trx (Fig 8B, arrow). The significance of the Trx/14-3-3 complex in the ROT environment remains unclear, but this study is unique in illuminating the native arrangements of the endogenous complexes involving the trio ASK1, Trx and 14-3-3 proteins. As panel C indicates, pre-incubating cells with 200 pM recombinant secreted Klotho for at least 40 min prior to ROT treatment thwarted ASK1 dissociation from the complex (Fig 8C, arrow). There is a slight shift of the entire complex to the acid end, which appears consistent in the assays, but the *p*I accuracy and drift of the system is within this range. Furthermore, the macromolecular complex is quite large and precise focusing is not expected beyond a few tenths of a *p*I unit. It should be noted that other prominent individual signals belonging to either one of the three proteins were consistently found spanning the entire *p*I profile, suggestive of secondary interactions with other proteins comprising the ASK1 signaling complex or alternative forms and complexes with other molecules. These interactions would not have been revealed based on the denaturing and often reducing environments of SDS-PAGE Western blot analysis.

**Fig 9:**
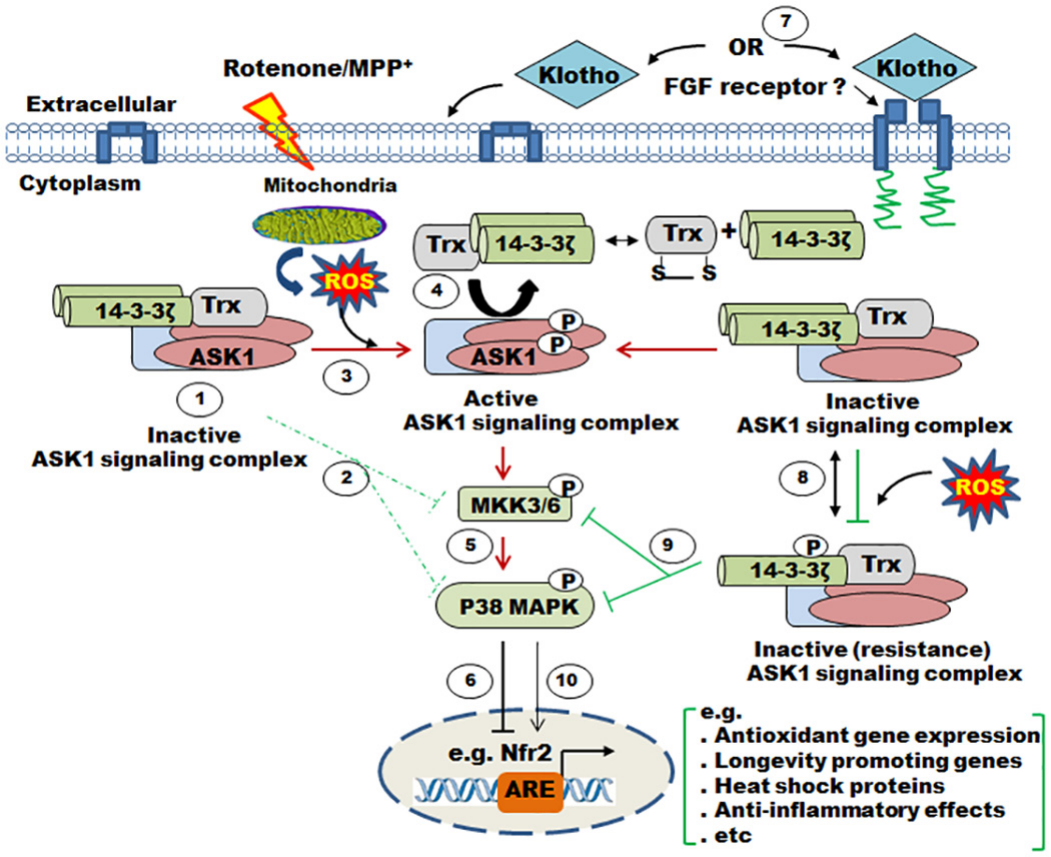
Model describing oxidative stress response in Klotho responsive cells and tissues. (*1–2*) In normal unstressed cells and tissues, 14-3-3ζ and thioredoxin cooperate with other cytosolic proteins to inhibit the ASK1 signaling complex with concomitant downstream reduction in MAPKs phosphorylation level. (*3–6*) In an oxidizing environment generated by reactive oxygen species (ROS) via mitochondrial electron transport chain dysfunction induced by rotenone, ASK1 dissociates from thioredoxin/14-3-3ζ, becomes phosphorylated (activating ASK1 signaling compex) and, in turn, phosphorylates downstream MAPKs, including p38. These cascades of events inhibit nuclear translocation of transcription factors such as Nrf2, leading to suppression of antioxidant and longevity-promoting genes. Puzzling is our observation that 14-3-3ζ and thioredoxin remained partially bound. (*7–10*) By contrast, 14-3-3ζ interaction with the ASK1 signaling complex is consolidated by Klotho-dependent phosphorylation of the 14-3-3ζ at Ser-58, promoting monomer formation; 14-3-3ζ monomerization may increase its interaction with the inhibitory ASK1 by forming a stable complex. This activity is expected to facilitate Nrf2 nuclear localization promoting expression of antioxidant and longevity-promoting genes.

Given that 14-3-3ζ is required for Klotho’s anti-oxidant activity via the ASK1/p38 MAPK pathway, Fig 9 summarizes Klotho’s regulatory role in this pathway. In the absence of Klotho, the ASK1 signaling complex is subject to oxidation and dissociation, in response to ROS. This culminates in auto-phosphorylation of ASK1 [17] triggering downstream activation events via p38 MAPK, and eventual suppression of antioxidant and longevity-promoting genes. By contrast, Klotho stimulation, whether by acting directly or through a receptor phosphorylates 14-3-3ζ at ser-58, increases the monomer forms, and stabilizes its interaction with the ASK1 signaling complex. This signaling activity is felt downstream of the p38 MAPK, facilitating Nrf2 nuclear localization and promoting expression of antioxidant and longevity-promoting genes.

## Discussion

We developed *in vitro* assays using HEK 293 cells to study Klotho’s mitigating effect on signaling in the ASK1/p38 MAPK, stress-response pathway. The procedure employs an immunoprecipitation regimen to measure Trx-mediated changes in ASK1 signaling induced by rotenone (ROT) treatment in the presence or absence of secreted Klotho. The assay principle is based on existing knowledge that Trx reversibly interacts with ASK1 to regulate its dissociation from the ASK1 signaling complex [14,17]. By using this assay system to detect bound Trx by Western blot, we produce strong evidence that Klotho stabilizes the interaction of Trx with ASK1 partly by consolidating 14-3-3ζ interaction in the ASK1/Trx/14-3-3ζ tripartite assembly through posttranslational modification. The protection of the complex against oxidative dissociation was maintained downstream of the signaling complex to the p38 MAPK phosphorylation level; phosphorylation of p38 was the actual marker of stress susceptibility. The present cellular data, therefore, complement our recent studies in the liver on Klotho, where a significant increase in the steady-state activation of the ASK1/p38 pathway was associated with the Klotho knockout (*kl/kl*) mice, while the converse result was observed in the Klotho overexpression strains [16]. The *kl/kl* mice are models for accelerated aging [3,30], while the transgenic strains exhibit increased lifespan [3,30]. This suggests that in addition to the previously reported effects of Klotho on IGF signaling [5] the anti-aging effect of Klotho is, in part, mediated through the regulation of anti-oxidant proteins and stress signaling through multi-protein complexes.

We demonstrate that Klotho’s suppressive effect on p38 MAPK activation is comparable to that observed using *N*-Acetyl cysteine (NAC), an established chemical antioxidant, though the two may operate via different mechanisms. Whereas we propose that the mechanism of attenuation of p38 MAPK activity by Klotho treated HEK 293 cells is dependent on both Trx and 14-3-3ζ acting cooperatively to shield the ASK1 from oxidative dissociation, NAC generally enacts resistance to oxidative stress by scavenging ROS. Indeed, our results show that NAC’s protective effect on the Trx/ASK1 complex dissociation was negligibly compromised by the 14-3-3ζ depletion whereas Klotho has an equivalent or greater anti-oxidant effect that depended solely on 14-3-3ζ expression levels. Strikingly, our data further reveal that Klotho mediated phosphorylation of endogenous 14-3-3ζ (Ser-58) promotes monomer formation; we infer that it is the monomer influx that enhances 14-3-3ζ binding to Trx and subsequent stabilization of the signaling complex. This is in contrast to a popularly held believe that 14-3-3 dimers are the predominant phospho-protein interacting species. This proposition is further supported by our finding that Trx interacts more efficiently with artificially expressed dimer-deficient 14-3-3ζ mutant.

We were puzzled that phosphorylation of 14-3-3ζ (Ser-58) coincides with the stability of the ASK1 signaling complex. Our finding may indicate a new twist in cellular relevance of 14-3-3ζ phosphorylation and changes in cell survival. It has been reported that the binding of 14-3-3 proteins to the carboxyl (C) terminus of ASK1 is phosphorylation-dependent and occurs through Ser-967 [31]. But its dissociation from ASK1 and subsequent activation of the latter is not straight forward. At least two mechanisms have been put forward to date: that de-phosphorylation of the Ser-967 by the protein phosphatase calcineurin leads to ASK1 release from 14-3-3 proteins [26]; more recently, it was reported that an oxidant such as H_2_O_2_ promotes phosphorylation of overexpressed 14-3-3ζ at Ser-58 resulting in its dissociation from ASK1 [32]. Our combined data, however, show that Klotho’s paradoxical effects on 14-3-3ζ phosphorylation (Ser-58), results in: 1) increasing 14-3-3ζ monomer levels; 2) protection against Trx/ASK1 complex dissociation; and, 3) reducing the phosphorylation level of p38 MAPK. This is corroborated by analyses of brain lysates for basal levels of 14-3-3ζ phosphorylation (Ser-58), 14-3-3ζ monomer levels, and Trx/ASK1 complex level in our Klotho mouse models (reported separately as companion to this paper); all relative levels of the Trx/ASK1 complex, and Ser-58 mediated monomerization, depended on steady-state Klotho expression in each model. In summary, it is remarkable that although 14-3-3ζ Ser-58 phosphorylation was shown to release ASK1 to activate apoptotic pathways [32], our present data from Klotho treated cells and separate data on mouse model brains suggest that endogenously phosphorylated 14-3-3ζ (Ser-58) has regulatory effects opposite to those expected and is associated with reduced oxidative stress. These diametrically opposed effects possibly stem from the fact that prior experimentation depended on overexpressed recombinant 14-3-3 in cell lines, whereas we were concerned with endogenous activity. Indeed, overexpression of recombinant 14-3-3 in our various stress assays mostly lead to results opposite than expected (data not shown). Different protein kinases and phosphatases associated with the regulation of 14-3-3 proteins have been described elsewhere [32–34]; it is yet to be confirmed whether the same and/or different kinases and phosphatases are turned on or off by soluble Klotho. Towards these ends, we have initiated kinome wide siRNA screening of mammalian cell lines to identify kinases whose loss of function could mediate Klotho’s effect [35].

Existing models describing regulation of ASK1 activation propose that Trx interaction with the amino (N) terminus of ASK1 is redox-sensitive, which requires the two critical Trx active site cysteine (Cys32/35) residues to be in a reduced state to facilitate the protein-protein interaction [17,36]. Nevertheless, once formed, the Trx/ASK1 complex is subject to ROS-based oxidation and subsequent release of ASK1 from it captors [17,36]. While an amino acid substitution of either one of the two catalytic Cys residues does not abolish Trx/ASK1 interaction, the complex formed was found to be resistant to ROS-induced dissociation [29,37]. This suggests an alternate interpretation of the Trx/ASK1 interaction, specifically, that covalent inter-molecular bridges may be occurring between the proteins of the signaling complex. Indeed, our IP and non-reducing 2D Western blot analyses of the signaling complex support the occurrence of a mixed disulfide bridge. Moreover, our data suggest that accessibility to the Trx/ASK1 mixed disulfide bridge could be restricted due to structural rearrangement orchestrated by interacting molecules such as the 14-3-3 proteins, which are known phospho-binding proteins with possible scaffolding functions [38,39]. Klotho-mediated, phosphorylation (Ser-58)-induced monomerization of 14-3-3ζ promotes 14-3-3ζ binding to Trx and/or ASK1 within the complex, which in turn may shield and stabilize the Trx/ASK1 interaction in the presence of ROS. This is supported by the fact that knockdown of 14-3-3ζ expression by siRNA antagonizes its effect on the Trx/ASK1 complex formation. A ligand-mediated protection against oxidant-induced Trx/ASK1 complex dissociation is not unheard of in other contexts. For example, the HIV protein Nef was shown to associate with ASK1 and inhibit its oxidant-induced apoptosis in Jurkat cells, and was subsequently traced to the inhibition of Trx/ASK1 complex dissociation [40]. However, it is yet to be known whether a relationship exists between Nef and effectors of Klotho activation.

In conclusion, this study demonstrates that secreted Klotho can initiate anti-oxidant signaling events when added exogenously into cultured cells. Whereas the question of whether FGF receptors are involved in this process still remains, silencing of 14-3-3ζ expression antagonizes the Klotho anti-oxidant signaling effect, suggestive of integral involvement of 14-3-3 members in this cascade. Klotho mediated phosphorylation of 14-3-3ζ (Ser-58) is dynamically regulated in a cell, implying that the kinases and phosphatases involved in this process may be part of a broader signaling cascade. There is no doubt our accumulated data show that secreted Klotho, whether by acting directly or through a receptor, initiates cellular events culminating in 14-3-3ζ-dependent protection of the inhibitory ASK1 signaling complex and subsequent attenuation of the downstream p38 MAPK activation leading to oxidative stress resistance. At the same time, disruption of Klotho expression would lead to uncontrolled activation of the ASK1 signaling complex permitting sustained activation of the p38 MAPK and chronic oxidative stress. Although much remains to be learned regarding Klotho’s involvement in ASK1/p38 MAPK stress response pathways, our data strongly suggest Klotho activity regulates ASK1 signaling complex by stabilizing 14-3-3ζ interaction with the complex, culminating in downstream reduction of MAPKs activation levels. In conjunction with our animal study reported separately as companion to this paper we propose that Klotho-mediated protection of the inhibitory ASK1 signaling complex represents one mechanism for safeguarding midbrain dopaminergic neurons against ROS-induced oxidative damage; this study complements several recent reports implicating Klotho in defense mechanism against brain degeneration [8–11] and yields a putative molecular mechanism whereby Klotho exhibits its cellular and tissue effects. In addition, this study introduces new interactive model describing regulation of the ASK1 signaling complex by 14-3-3 proteins. Higher plasma klotho concentrations were associated with a reduction of the aging-related cognitive decline in large longitudinal cohort [41]. Strikingly, the allele variants rs9536314 (F352V), and rs9527025 (C370S), of the *KLOTHO* gene *KL*, forming the haplotype “KL-VS”, have been associated to increase cognitive abilities such as memory [42], verbal and non-verbal [43], and gray matter volumes in aging human brains [44]. Two other variants positioned in the 5′ end of the *KL* gene, rs398655 and rs562020, were associated with enhanced cognitive abilities unrelated to KL-VS [45]. The effect of Klotho in the brains of the overexpression models appears to be neuro-protective and prevents the loss of dopaminergic neurons [46]. From a structural standpoint, such a Klotho-induced preserved cognition underlies integrity of brain plasticity and the potential of neurons to change their synaptic connections [47]. The lengthening of axons, sprouting of collateral ramifications, and remodeling allow the dwelling of new synapses, new cognitive and behavioral operations [46]. The Klotho-induced jump in brain resistance to high levels of oxidative stress and pathogenic proteins, seems to be a major enhancer of normal cognition, independent of age.

## Supporting Information

S1 Fig Dissociation of thioredoxin (Trx) from complexes immunoprecipitated (IP) with 14-3-3zeta. (TIF)

S1 Table Digitized numerical values of Western blot of Trx/ASK1 complex levels in Klotho/NAC treated HEK 293. (XLSX)

S2 Table Digitized numerical values of Western blot of p-38 MAPK activation levels in Klotho/NAC treated HEK 293. (XLSX)

S3 Table Digitized numerical values of Western blot of Klotho-induced monomerization of 14-3-3zeta in HEK 293. (XLSX)

S4 Table Digitized numerical values of Western blot of levels of Trx bound to recombinant WT and dimer-deficient (Dm) 14-3-3zeta. (XLSX)

S5 Table Digitized numerical values of Western blot of Klotho-induced increased interaction of Trx with 14-3-3zeta. (XLSX)

## References

[1] Lakowski B, Hekimi S (1998) The genetics of caloric restriction in Caenorhabditis elegans. Proc Natl Acad Sci U S A 95: 13091–13096. 9789046 10.1073/pnas.95.22.13091 PMC23719

[2] Papaconstantinou J (1994) Unifying model of the programmed (intrinsic) and stochastic (extrinsic) theories of aging. The stress response genes, signal transduction-redox pathways and aging. Ann N Y Acad Sci 719: 195–211. 8010593 10.1111/j.1749-6632.1994.tb56829.x

[3] Kuro-o M, Matsumura Y, Aizawa H, Kawaguchi H, Suga T, Utsugi T, et al (1997) Mutation of the mouse klotho gene leads to a syndrome resembling ageing. Nature 390: 45–51. 10.1038/36285 9363890

[4] Kuro-o M (2006) Klotho as a regulator of fibroblast growth factor signaling and phosphate/calcium metabolism. Curr Opin Nephrol Hypertens 15: 437–441.; 00041552-200607000-00013 [pii]. 16775459 10.1097/01.mnh.0000232885.81142.83

[5] Kurosu H, Ogawa Y, Miyoshi M, Yamamoto M, Nandi A, Rosenblatt KP, et al (2006) Regulation of fibroblast growth factor-23 signaling by klotho. J Biol Chem 281: 6120–6123. C500457200 [pii]; 10.1074/jbc.C500457200 16436388 PMC2637204

[6] Wolf I, Levanon-Cohen S, Bose S, Ligumsky H, Sredni B, Kanety H, et al (2008) Klotho: a tumor suppressor and a modulator of the IGF-1 and FGF pathways in human breast cancer. Oncogene 27: 7094–7105. onc2008292 [pii];10. 18762812 10.1038/onc.2008.292

[7] Duce JA, Podvin S, Hollander W, Kipling D, Rosene DL, Abraham CR (2008) Gene profile analysis implicates Klotho as an important contributor to aging changes in brain white matter of the rhesus monkey. Glia 56: 106–117. 10.1002/glia.20593 17963266

[8] Chen CD, Sloane JA, Li H, Aytan N, Giannaris EL, Zeldich E, et al (2013) The antiaging protein Klotho enhances oligodendrocyte maturation and myelination of the CNS. J Neurosci 33: 1927–1939. 33/5/1927 [pii]; 10.1523/JNEUROSCI.2080-12.2013 23365232 PMC3711388

[9] Shin EJ, Chung YH, Le HL, Jeong JH, Dang DK, Nam Y, et al (2015) Melatonin attenuates memory impairment induced by Klotho gene deficiency via interactive signaling between MT2 receptor, ERK, and Nrf2-related antioxidant potential. Int J Neuropsychopharmacol 18 pyu105 [pii]; 10.1093/ijnp/pyu105 PMC4438546 25550330

[10] Wang X, Sun Z (2010) RNAi silencing of brain klotho potentiates cold-induced elevation of blood pressure via the endothelin pathway. Physiol Genomics 41: 120–126. 00192.2009 [pii]; 10.1152/physiolgenomics.00192.2009 20086041 PMC2853900

[11] Zeldich E, Chen CD, Colvin TA, Bove-Fenderson EA, Liang J, Tucker Zhou TB, et al (2014) The neuroprotective effect of Klotho is mediated via regulation of members of the redox system. J Biol Chem 289: 24700–24715. M114.567321 [pii]; 10.1074/jbc.M114.567321 25037225 PMC4148892

[12] Yamamoto M, Clark JD, Pastor JV, Gurnani P, Nandi A, Kurosu H, et al (2005) Regulation of oxidative stress by the anti-aging hormone klotho. J Biol Chem 280: 38029–38034. M509039200 [pii]; 10.1074/jbc.M509039200 16186101 PMC2515369

[13] Hsieh CC, Papaconstantinou J (2004) Akt/PKB and p38 MAPK signaling, translational initiation and longevity in Snell dwarf mouse livers. Mech Ageing Dev 125: 785–798. S0047-6374(04)00169-1 [pii]; 10.1016/j.mad.2004.07.008 15541773

[14] Hsieh CC, Papaconstantinou J (2006) Thioredoxin-ASK1 complex levels regulate ROS-mediated p38 MAPK pathway activity in livers of aged and long-lived Snell dwarf mice. FASEB J 20: 259–268. 20/2/259 [pii]; 10.1096/fj.05-4376com 16449798 PMC1479092

[15] Sohal RS, Agarwal S, Sohal BH (1995) Oxidative stress and aging in the Mongolian gerbil (Meriones unguiculatus). Mech Ageing Dev 81: 15–25. 004763749401578A [pii]. 7475349 10.1016/0047-6374(94)01578-a

[16] Hsieh CC, Kuro-o M, Rosenblatt KP, Brobey R, Papaconstantinou J (2010) The ASK1-Signalosome regulates p38 MAPK activity in response to levels of endogenous oxidative stress in the Klotho mouse models of aging. Aging (Albany NY) 2: 597–611. 100194 [pii]. 20844314 10.18632/aging.100194 PMC2984608

[17] Saitoh M, Nishitoh H, Fujii M, Takeda K, Tobiume K, Sawada Y, et al (1998) Mammalian thioredoxin is a direct inhibitor of apoptosis signal-regulating kinase (ASK) 1. EMBO J 17: 2596–2606. 10.1093/emboj/17.9.2596 9564042 PMC1170601

[18] Tobiume K, Matsuzawa A, Takahashi T, Nishitoh H, Morita K, Takeda K, et al (2001) ASK1 is required for sustained activations of JNK/p38 MAP kinases and apoptosis. EMBO Rep 2: 222–228. 10.1093/embo-reports/kve046 11266364 PMC1083842

[19] Tzivion G, Luo Z, Avruch J (1998) A dimeric 14-3-3 protein is an essential cofactor for Raf kinase activity. Nature 394: 88–92. 10.1038/27938 9665134

[20] Aitken A, Baxter H, Dubois T, Clokie S, Mackie S, Mitchell K, et al. (2002) Specificity of 14-3-3 isoform dimer interactions and phosphorylation. Biochem Soc Trans 30: 351–360. 12196094 10.1042/bst0300351

[21] Porter GW, Khuri FR, Fu H (2006) Dynamic 14-3-3/client protein interactions integrate survival and apoptotic pathways. Semin Cancer Biol 16: 193–202. S1044-579X(06)00027-7 [pii]; 10.1016/j.semcancer.2006.03.003 16697216

[22] Woodcock JM, Murphy J, Stomski FC, Berndt MC, Lopez AF (2003) The dimeric versus monomeric status of 14-3-3zeta is controlled by phosphorylation of Ser58 at the dimer interface. J Biol Chem 278: 36323–36327. 10.1074/jbc.M304689200; M304689200 [pii]. 12865427

[23] Xiao B, Smerdon SJ, Jones DH, Dodson GG, Soneji Y, Aitken A, et al (1995) Structure of a 14-3-3 protein and implications for coordination of multiple signalling pathways. Nature 376: 188–191. 10.1038/376188a0 7603573

[24] Choi JE, Hur W, Jung CK, Piao LS, Lyoo K, Hong SW, et al (2011) Silencing of 14-3-3zeta over-expression in hepatocellular carcinoma inhibits tumor growth and enhances chemosensitivity to cis-diammined dichloridoplatium. Cancer Lett 303: 99–107. S0304-3835(11)00040-1 [pii]; 10.1016/j.canlet.2011.01.015 21334806

[25] Sturm N, Jortzik E, Mailu BM, Koncarevic S, Deponte M, Forchhammer K, et al (2009) Identification of proteins targeted by the thioredoxin superfamily in Plasmodium falciparum. PLoS Pathog 5: e1000383 10.1371/journal.ppat.1000383 19360125 PMC2660430

[26] Liu Q, Wilkins BJ, Lee YJ, Ichijo H, Molkentin JD (2006) Direct interaction and reciprocal regulation between ASK1 and calcineurin-NFAT control cardiomyocyte death and growth. Mol Cell Biol 26: 3785–3797. 26/10/3785 [pii]; 10.1128/MCB.26.10.3785-3797.2006 16648474 PMC1489013

[27] Liu Y, Yin G, Surapisitchat J, Berk BC, Min W (2001) Laminar flow inhibits TNF-induced ASK1 activation by preventing dissociation of ASK1 from its inhibitor 14-3-3. J Clin Invest 107: 917–923. 10.1172/JCI11947 11285311 PMC199579

[28] Hansen RE, Winther JR (2009) An introduction to methods for analyzing thiols and disulfides: Reactions, reagents, and practical considerations. Anal Biochem 394: 147–158. S0003-2697(09)00538-7 [pii]; 10.1016/j.ab.2009.07.051 19664585

[29] Liu Y, Min W (2002) Thioredoxin promotes ASK1 ubiquitination and degradation to inhibit ASK1-mediated apoptosis in a redox activity-independent manner. Circ Res 90: 1259–1266. 12089063 10.1161/01.res.0000022160.64355.62

[30] Kurosu H, Yamamoto M, Clark JD, Pastor JV, Nandi A, Gurnani P, et al (2005) Suppression of aging in mice by the hormone Klotho. Science 309: 1829–1833. 1112766 [pii]; 10.1126/science.1112766 16123266 PMC2536606

[31] Zhang L, Chen J, Fu H (1999) Suppression of apoptosis signal-regulating kinase 1-induced cell death by 14-3-3 proteins. Proc Natl Acad Sci U S A 96: 8511–8515. 10411906 10.1073/pnas.96.15.8511 PMC17547

[32] Zhou J, Shao Z, Kerkela R, Ichijo H, Muslin AJ, Pombo C, et al (2009) Serine 58 of 14-3-3zeta is a molecular switch regulating ASK1 and oxidant stress-induced cell death. Mol Cell Biol 29: 4167–4176. MCB.01067-08 [pii]; 19451227 10.1128/MCB.01067-08 PMC2715813

[33] Powell DW, Rane MJ, Chen Q, Singh S, McLeish KR (2002) Identification of 14-3-3zeta as a protein kinase B/Akt substrate. J Biol Chem 277: 21639–21642. 10.1074/jbc.M203167200; M203167200 [pii]. 11956222

[34] Powell DW, Rane MJ, Joughin BA, Kalmukova R, Hong JH, Tidor B, et al (2003) Proteomic identification of 14-3-3zeta as a mitogen-activated protein kinase-activated protein kinase 2 substrate: role in dimer formation and ligand binding. Mol Cell Biol 23: 5376–5387. 12861023 10.1128/MCB.23.15.5376-5387.2003 PMC165733

[35] Komurov K, Padron D, Cheng T, Roth M, Rosenblatt KP, White MA (2010) Comprehensive mapping of the human kinome to epidermal growth factor receptor signaling. J Biol Chem 285: 21134–21142. M110.137828 [pii]; 10.1074/jbc.M110.137828 20421302 PMC2898331

[36] Holmgren A (1989) Thioredoxin and glutaredoxin systems. J Biol Chem 264: 13963–13966. 2668278

[37] Nadeau PJ, Charette SJ, Toledano MB, Landry J (2007) Disulfide Bond-mediated multimerization of Ask1 and its reduction by thioredoxin-1 regulate H(2)O(2)-induced c-Jun NH(2)-terminal kinase activation and apoptosis. Mol Biol Cell 18: 3903–3913. E07-05-0491 [pii]; 10.1091/mbc.E07-05-0491 17652454 PMC1995733

[38] Aitken A, Jones D, Soneji Y, Howell S (1995) 14-3-3 proteins: biological function and domain structure. Biochem Soc Trans 23: 605–611. 8566426 10.1042/bst0230605

[39] Aitken A (1996) 14-3-3 and its possible role in co-ordinating multiple signalling pathways. Trends Cell Biol 6: 341–347. 0962-8924(96)10029-5 [pii]. 15157431 10.1016/0962-8924(96)10029-5

[40] Geleziunas R, Xu W, Takeda K, Ichijo H, Greene WC (2001) HIV-1 Nef inhibits ASK1-dependent death signalling providing a potential mechanism for protecting the infected host cell. Nature 410: 834–838. 10.1038/35071111; 35071111 [pii]. 11298454

[41] Shardell M, Semba RD, Rosano C, Kalyani RR, Bandinelli S, Chia CW, et al (2015) Plasma Klotho and Cognitive Decline in Older Adults: Findings From the InCHIANTI Study. J Gerontol A Biol Sci Med Sci. glv140 [pii]; 10.1093/gerona/glv140 PMC5007737 26297657

[42] Dubal DB, Yokoyama JS, Zhu L, Broestl L, Worden K, Wang D, et al (2014) Life extension factor klotho enhances cognition. Cell Rep 7: 1065–1076. S2211-1247(14)00287-3 [pii]; 10.1016/j.celrep.2014.03.076 24813892 PMC4176932

[43] Deary IJ, Harris SE, Fox HC, Hayward C, Wright AF, Starr JM, et al (2005) KLOTHO genotype and cognitive ability in childhood and old age in the same individuals. Neurosci Lett 378: 22–27. S0304-3940(04)01520-4 [pii]; 10.1016/j.neulet.2004.12.005 15763166

[44] Yokoyama JS, Sturm VE, Bonham LW, Klein E, Arfanakis K, Yu L, et al (2015) Variation in longevity gene KLOTHO is associated with greater cortical volumes. Ann Clin Transl Neurol 2: 215–230. 10.1002/acn3.161 25815349 PMC4369272

[45] Mengel-From J, Soerensen M, Nygaard M, McGue M, Christensen K, Christiansen L (2015) Genetic Variants in KLOTHO Associate With Cognitive Function in the Oldest Old Group. J Gerontol A Biol Sci Med Sci. glv163 [pii]; 10.1093/gerona/glv163 PMC4978356 26405063

[46] Foster PP, Rosenblatt KP, Kuljis RO (2011) Exercise-induced cognitive plasticity, implications for mild cognitive impairment and Alzheimer's disease. Front Neurol 2: 28 10.3389/fneur.2011.00028 21602910 PMC3092070

[47] Ashford JW, Jarvik L (1985) Alzheimer's disease: does neuron plasticity predispose to axonal neurofibrillary degeneration? N Engl J Med 313: 388–389. 4010760

