# Supplementary figures and images for: Klotho Regulates 14-3-3ζ Monomerization and Binding to the ASK1 Signaling Complex in Response to Oxidative Stress
Source: PLoS One. 2015 Oct 30;10(10):e0141968. doi: 10.1371/journal.pone.0141968 (PMC4627807; doi:10.1371/journal.pone.0141968)

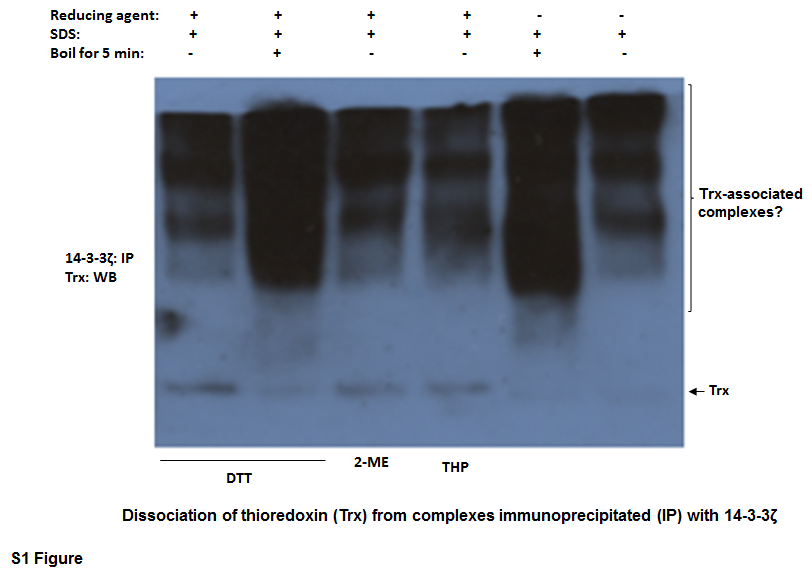

Supplement: S1 Fig — (TIF) [file pone.0141968.s001.tif]
